# Leadership Education at a Crossroads: Adapting the ILA General Principles for a Changing World

**DOI:** 10.1002/yd.70009

**Published:** 2025-08-22

**Authors:** Dennis C. Roberts

**Affiliations:** ^1^ New Dimensions in Education Wilmette Illinois

## Abstract

ILA *General Principles for Leadership Programs* were originally conceived to advocate approaches to leadership cultivation that would increase visibility, encourage greater cooperation, and be applicable across diverse organizations and cultural contexts. Subsequent to their release as a concept paper, the *General Principles* have been disseminated and tested in a variety of ways and are now promoted as a model for coherent and comprehensive leadership program design and implementation. While the *General Principles* achieved some success, this concluding article proposes that economic, social, and political changes must be considered when using the *General Principles* and that it is essential that leadership educators commit to greater consensus and coherence in their shared work.

## 1 Introduction

The International Leadership Association's (ILA) *General Principles for Leadership Programs* (ILA 2021) emerged with the input of many dedicated scholars and practitioners of leadership learning. This *New Directions for Student Leadership* issue is dedicated to explaining who worked on the *General Principles for Leadership Programs* (ILA 2021) and what motivated them. It then took the ideas in the foundational document to deeper levels by providing examples or proposals for their use.

The authors of the previous articles in this issue are all deeply informed about and committed to the *General Principles for Leadership Programs* (ILA 2021). Their deep exploration of the context, conceptual framework, content, learning and metrics, outcomes, and assessment for leadership initiatives reveals the necessity of alignment. Although the five areas were not originally proposed as hierarchical, the practical matter is that, to achieve alignment, planners need to start with context and then move to conceptual framework. With these as a foundation, the determination of content, learning, and metrics, outcomes, and assessment then unfolds in a mutually informing way that ensures that these three broad areas line up with one another.

As a simple example, selection of content will make the most positive impact if the institutional or organizational climate is understood and the content meaningfully derived from the vision outlined in the context and conceptual framework. The content, uniqueness of learners, and the measurement of outcomes and success are then woven in and through each other to create a fabric that will result in leadership being central, its portrayal coherent, and for it to be a catalyst for cooperation.

With these insights in mind, this article moves to what has been achieved through promoting the *General Principles for Leadership Programs* (ILA 2021) and then the all‐important question of what has changed about contemporary political, economic, and social conditions in the current day and what implications current trends have for the continual improvement and use of the *General Principles for Leadership Programs* (ILA 2021).

## 2 What Has Been the Outcome?

The intent of contributors to the ILA *General Principles for Leadership Programs* (ILA 2021) was to foster a conversation about how leadership learning practices could be enhanced by distributing the document widely and encouraging application and modification of its propositions over time. While presentations have been offered at numerous conferences, several webinars have been offered to ILA members, and some publications have spread the word, the sobering realization is that there is so much more that can be done to champion the *General Principles for Leadership Programs* (ILA 2021) among leadership educators—hopefully surging throughout ILA stakeholders and beyond.

Dissemination and use of the *General Principles for Leadership Programs* (ILA 2021) is complicated by several factors. Leadership educators are frequently consumed by their roles and are therefore focused on doing the best they can with limited time and resources. There has also been reluctance among some leadership programs to relinquish what is sometimes perceived to be a unique niche or specialty in fostering leadership learning. The proliferation of leadership programs across institutions has resulted in wide variations in resources dedicated to leadership learning, a dynamic that makes comparison across programs challenging. One of the primary contributing factors could be that the commitment to ‘modeling’ for leadership programs includes multiple attempts such as those that follow.

Long before ILA began its journey with the *General Principles for Leadership Programs* (ILA 2021), the Council for the Advancement of Standards (CAS 2019) had a framework and standards in place for use by leadership educators largely working out of the extra‐ or cocurricular area of most higher education institutions. More recently, the Leadership for Public Purpose Classification (American Council on Education n.d.) emerged to provide a model and invited applications to colleges and universities making an institution‐wide commitment to foster leadership for public purpose. The first class of designees for Leadership for Public Purpose was identified in 2024.

Most recently, leadership educators convened in 2024 to explore gaps in leadership, vision, future knowledge needs, and shape the future of leadership research. The Future Forward Leadership Summit (2024) included the generation of key insights related to construct proliferation; measurement; leadership and diversity, equity, inclusion, and belonging; the changing nature of work; stress; sustainability; development; and research to practice related to leadership. While the superficial reaction to multiple approaches might suggest overlap or competition, the previous three examples, in synergy with the ILA *General Principles for Leadership Programs* (ILA 2021), jointly advocate comprehensiveness and cohesiveness, potentially resulting in complementary efforts to cultivate leadership more deeply and broadly.

The ILA Committee on the Advancement of Leadership Programs deliberately sought synergies with other initiatives, understanding that the outcome of leadership capacity building had fallen short (Kellerman 2018) and striving to enhance the study and practice of leadership for the benefit of all.

## 3 What Has Changed as We Look to the Future?

The way we conceptualize leadership has undergone significant transformation in the very short period from 2016 to the present. The shifts in economic, social, and political dynamics since the formulation and drafting of the *General Principles for Leadership Programs* (ILA 2021) are profound. The most significant implications of these changes include increasing competitive isolation and the decline of the effectiveness of soft power and knowledge diplomacy.

### 3.1 Economic, Social, and Political Shifts

The world today is grappling with a range of shifting dynamics that challenge notions of leadership many researchers and educators advocate. Three of the most notable changes involve the emergence of nationalism, authoritarianism, and isolationism as powerful global trends. These forces are reshaping how we approach leadership at both the national and international levels. The rise of competitive isolationism increasingly permeates international negotiations, placing economic gain against competitors at the center of relations rather than the pursuit of mutual interests.

This competitive isolationism arises from a complex set of variables that challenge the globalized systems in which leadership should flourish:

1. **Cultural Relativism and the Rejection of Colonialism**: The rejection of colonial domination by emerging Indigenous cultures has led to a more heterogeneous world. As these cultures assert their identities, they resist the imposition of foreign norms and governance structures, fostering a landscape of diverse national interests.
2. **Divisiveness of Identity Politics**: Increasingly, political discourse has been fractured by identity politics, where assertions of grievances often exacerbate divisions within societies. This fracturing undermines the collective cooperation needed for global leadership.
3. **Declining Trust in Institutions**: A significant challenge to leadership in the modern age is the widespread decline in trust accorded to institutions. Political systems, media outlets, and even academic and economic entities are facing growing skepticism, which impedes the capacity for effective governance and leadership.
4. **Disinformation**: The proliferation of partisan commercial media and social media has amplified the spread of disinformation. This distorted narrative not only confuses public understanding but also shapes political behavior in ways that exacerbate isolationist tendencies.

As a result, countries are increasingly turning inward, focusing on protecting jobs and cultures through measures like raising tariffs. However, as Bremmer (2018) argued, these protectionist policies often lead to retaliation from other nations, ultimately escalating tensions or prompting the relocation of production to more favorable regions.

### 3.2 Decline of Soft Power and Knowledge Diplomacy

The days of relying on soft power and knowledge diplomacy, as discussed by Knight (2022), seem to be in decline. The shift toward a more competitive, win/lose mentality, undermines the collaborative spirit that once underpinned international relations. This has fractured partnerships and eroded efforts toward addressing mutual, pan‐human concerns, such as climate change, global health, and economic inequality.

The loss of long‐standing conventions in diplomacy, economic cooperation, and educational practices necessitates a shift in leadership approaches. Therefore, leadership in and for the future must be oriented around adaptability, equity, and inclusion.

1. **Adapting to Change**: It is crucial to help citizens understand that survival in the modern world depends on their ability to adapt. Governments must demonstrate that they can evolve to meet the needs of their populations, particularly when led honestly and with a genuine commitment to the public good.
2. **Addressing Inequality**: Governments must also tackle inequality more effectively. The increasing disparity between the wealthy and the disadvantaged calls for leadership that strives to lift all boats—not just the yachts of the privileged few.
3. **Education and Retraining**: As global economies shift, education and retraining programs will become central to leadership development. Countries with large and diverse populations will face additional challenges in adapting to these changes, as noted by Bremmer (2018). However, the need for accessible and effective educational systems to meet these challenges is undeniable.

### 3.3 New Opportunities for Leadership Educators

These changes present both challenges and opportunities for leadership educators. As political, economic, and cultural barriers evolve, there is a need to replace scarcity‐based perspectives with a politics of abundance, as argued by Klein and Thompson (2025). Leadership educators must reframe leadership away from heroic and hierarchical models by placing priority on three commitments.

1. **Critiquing Traditional Leadership Models**: The traditional focus on heroic, top‐down leadership models must be critiqued and replaced with more inclusive and participatory approaches. These models should focus on mobilizing resources, both human and material, for the resolution of shared global challenges.
2. **Cultural Adaptation and Inclusivity**: Leadership education must be culturally adapted to reverse the exclusion and marginalization that have characterized much of traditional leadership discourse. This involves fostering leadership that is sensitive to local contexts while maintaining a global perspective on shared concerns.
3. **Addressing Desperation, Resentment, and Hope**: Leadership educators must also address the emotional and psychological landscape of many communities, which are grappling with feelings of desperation, resentment, and hope. An unsettled world order that redistributes economic and political influence is both an opportunity and a vulnerability. To move various entities toward hope will require abandoning individualism and embracing multi‐sector and multi‐cultural complexity that addresses shared challenges and fosters shared work.

The future of leadership hinges on its ability to engage with these complex emotions and provide a vision for a better, more inclusive world. As Khanna (2021) stated, “Civilizations of the past collapsed because they failed to adapt to the complexity they themselves created” (264).

The rise of nationalism, authoritarianism, and isolationism, combined with the decline of soft power and knowledge diplomacy, challenges contemporary notions of leadership and calls for a refinement and expansion in leadership educators’ thinking. The future of leadership learning relies on our ability to navigate this complexity and foster collaboration in the face of this adversity.

## 4 ILA *General Principles* Respond

The task is clear: the ILA *General Principles for Leadership Programs* (ILA 2021) captured the critical issues that were evident at the time. The rapid divergence from many of the common assumptions of that time now calls leadership educators to double‐down on some ideas while pressing forward to ensure the preservation and flourishing of leadership education as a whole. The most pressing opportunities include:

1. **New Opportunities for Relevance**: The changes we are witnessing today challenge leadership educators and capacity builders, but they also provide new opportunities to demonstrate their relevance and contribution. Past assumptions about political, economic, and cultural barriers need to be replaced by a politics of abundance that moves away from the scarcity perspectives gaining traction globally (Klein and Thompson 2025).
2. **Revising Leadership Models**: Continuing, and fortifying, the critique of the traditional, heroic, and hierarchical forms of leadership is essential. And this critique must include cultural adaptation, especially related to exclusion and marginalization, empowering leaders to mobilize human and other resources to resolve shared global challenges.
3. **Acknowledging the Leadership Industry's Role**: At this juncture, it is critical to acknowledge the reality that the leadership industry needs an approach, a framework, and principles to create synergies in research, theorizing, and practice. The promotion, use, and continuous adaptation of the ILA *General Principles for Leadership Programs* (ILA 2021) must respond to the ever‐evolving dynamics of the global landscape.
4. **Protecting Educators’ Roles**: At a time when public support for educators is in question and when those who benefit from the status quo actively seek to silence critical voices, it is essential to protect and defend the role of educators in general and leadership educators more specifically. Their contribution to shaping societies, systems, and individuals through education must be safeguarded.

### 4.1 Relevance of the General Principles to Contemporary Issues

The ILA *General Principles for Leadership Programs* (ILA 2021) holistically offer important guidance across five elements to build capacity in leadership—context, conceptual framework, content, learning, and assessment. Specific references from the *General Principles for Leadership Programs* (ILA 2021) that are directly relevant to the challenges of today's global landscape include:

- **Context**: “The need to understand global contexts and environmental drivers” (5).
- **Conceptual Framework**: “The ethical implications of power and the legitimacy of leadership” (6).
- **Equity and Justice**: “The need to advocate for equity, justice, and sustainability” (7).
- **Content**: “Understanding leadership and followership in global and local contexts” (9).
- **Toxic Leadership**: “Addressing how to recognize and deal with bad leadership” (9).
- **Global Dynamics**: “Understanding the global dynamics of the 21st century” (10).
- **Learning**: “The influence of social and cultural contexts on teaching and learning” (11–12).
- **Assessment**: “How program assessments consider their broader societal context” (14).

These references from the *General Principles* *for Leadership Programs* (ILA 2021) are only a few examples on which leadership educators are acting. These points warrant additional attention and focus, considering the current global environment.

### 4.2 ILA Member and Member Community Engagement

The ILA emerged as a grass‐roots movement of only a few scholars and advocates who sought to advance more purposeful and effective capacity building in leadership. Subsequent meetings involved ever‐growing numbers and diversity of interests, which contributed to its stature and attractiveness as a uniquely international organization. The growth and diversity of ILA was necessary to its sustainability, but this diversity also contributed to a dispersion of interests and the vulnerability of declining shared central interest.

The 17 member communities (https://ilaglobalnetwork.org/member‐communities/) are the best example of this. By sorting these communities into sectors, topics, purposes, and processes, a potential map of mutual interest emerges (see Table 1: ILA member communities).

**TABLE 1:** ILA member communities.

Area of primary focus	Member communities with stakeholder interest
Sectors	Arts and Leadership Business Leadership Healthcare Leadership Military/Uniformed Services Public Leadership Women and Leadership
Topics	Followership Leadership Scholarship Philosophy, Religion, and Worldview
Purposes	Ethics and Leadership Leadership for Peace Sustainability Leadership Trust and Leadership
Processes	AI and Emerging Technologies Leadership and Coaching Leadership Development Leadership Education

While the sorting proposed here might be different based on the views of those involved, beginning to see how the different groups all combine to form a mosaic of interest is the point. Each of these member communities has something to give and something to gain in being included in the conversation about the *General Principles for Leadership Programs* (ILA 2021).

Moving from a mosaic of interest to seeing how each of the ILA member communities could influence or be impacted by the *General Principles for Leadership Programs* (ILA 2021) provides a way to move from mere interest to investment. The member communities either have knowledge bases to inform, vested interest in the application, or could be transformed by advocating use of the *General Principles for Leadership Programs* (ILA 2021). An example from each of the four types of interests, within ILA and beyond, follows.

### 4.3 Sector Focus—Arts and Leadership

Two of the most important aspects where art and leadership are important as a sector are (1) how art inspires reflection on human experience and (2) how different actors in the arts express or personify leadership. Specifically in relation to leadership expression in the arts, attention has increasingly been given to leadership as an act of drawing out potential and contribution compared to previous eras where leadership may have been exercised as direction by an unquestionable authority. The different ways that art is expressed, and the variation in what is achieved, then offer examples as well as learning processes to cultivate effective leadership. The intersection of art and leadership is a source of content as well as a performative space to learn about leadership through observation and participation.

### 4.4 Topics Focus—Followership

As an example of topics of interest for those who research, theorize, and cultivate leadership, questions about followership have grown in their importance in recent years. Chaleff (2021) recounted the history and breadth of the study of followership and demonstrated that it has been integral to the understanding and cultivation of leadership for some time. Good followership and responsive leadership result in fluid input, contributions, and discernment of what is in the best interest of people and organizations. In passive or unassertive followership, bad leading is not called out, confronted, and resisted.

The *General Principles for Leadership Programs* specifically reference the need to “…help learners understand concepts such as leadership (formal and informal), followership, and context” (ILA 2021, 9) and how programs “…effectively contrast ‘good’ and ‘bad’ leadership practices through well documented real‐world examples” (ILA 2021, 11). Those dedicated to advancing the study and encouragement of responsible followership have a great deal to contribute to advancing positive leadership plus the attention to followership in the *Guiding Principles for Leadership Programs* (ILA 2021) is evidence that it is a critical area of inquiry and learning.

### 4.5 Purposes Focus—Trust and Leadership

As previously addressed in this article, current world conditions include divisiveness resulting from identity and class differences and a fundamental decline in trust across a breadth of organizations. Desperation and resentment within and across national borders are used as tools of power as various groups are set against each other through inflammatory rhetoric and action. Dividing people against each other, proclaiming others untrustworthy, and using disinformation to confuse are key to maintaining a position of power and control.

Understanding how these wedges are created and perpetuated will be central to the survival of checks and balances and accountability for those who seek to abuse leadership. The ILA *General Principles for Leadership Programs* call attention to these concerns by asking, “How does the program address the ethical complexities (e.g., good vs. bad; morality vs. immorality, moral absolutism vs. moral relativism, character, virtue, social justice, efficiency, moral reasoning, and constitutional rights) of inclusive leadership in action?” (ILA 2021, 11).

### 4.6 Processes Focus—Leadership Education

Knowing that insights on both content and processes of learning are being refined results in leadership educators needing to ask questions that are raised in the *General Principles for Leadership Programs*, such as, ʻʻIn what ways is self‐awareness fostered in participants as a foundation for ongoing and life‐long learning in leadership?’ or, “How does the program address learners' characteristics (i.e., identity development, culture, life experience, and comfort in learning)?” (ILA 2021, 10).

While these questions call for research to refine approaches to effective learning, they also require anyone directly involved in teaching leadership to be humble in their strategies—constantly reading, seeking input, and adjusting as required to the nature of learners. At the center of responding to learner characteristics is the question of readiness. The *General Principles for Leadership Programs* ask, “What instructional strategies are developmentally appropriate for the learners in the leadership program?” (ILA 2021, 13), which may be one of the most difficult questions for leadership educators.

Recognizing the mutual concerns that are currently expressed about the purposes and quality of leadership in multiple sectors and places around the world, the opportunity that lies ahead is for ILA to foster consensus about the shared work of leadership cultivation. Those who drafted the ILA *General Principles for Leadership Programs* (ILA 2021) and continue the work through the Committee on the Advancement of Leadership Programs, believed this was a necessary and achievable goal.

## 5 Moving Forward

The contributors to this *New Directions for Student Leadership* issue are only part of a much larger number of visionaries who seek to advance the study and practice of leadership. As contributors, many being members of the ILA Committee on the Advancement of Leadership Programs, they sought to inform the broader leadership learning community through a variety of means, with this issue providing a more comprehensive and readily available resource in the literature. This issue also confirms that the ILA *General Principles for Leadership Programs* (ILA 2021) are no longer provisional. The original draft, which referenced them as a “concept paper,” has changed. Although recognizing the need to revise them over time, the ideas and questions that are included in the document provide important modeling for use in multiple sectors and settings.

This issue recognized that 2025 is very different than the date of dissemination of the *General Principles for Leadership Programs* (ILA 2021). Recognizing this and adapting the *General Principles* to this new context, including the commitment to a 2025 revision, will help leadership scholars, educators, and those who practice leadership in various sectors.

### 5.1 Ways the ILA General Principles Are Being Applied

Three initiatives were taken to support the application of the *General Principles for Leadership Programs* (ILA 2021) in practice. Pilots with partnering higher education institutions included self‐study at the campus level, using the questions to probe for areas of strength and possible improvement. These self‐studies were then complemented by expert consultants who reviewed the self‐studies and offered outside perspective as a stimulus for continuous improvement. Lastly, Committee on the Advancement of Leadership Program members continue to engage with individuals and entities inside ILA as well as outside to coordinate and integrate the shared work of improving leadership capacity.

### 5.2 Perspective as a Source of Influence

Those committed to improving leadership must be attentive to how leadership is cultivated but also consider the perspective they bring to this work. The dramatic shifts that are seen in the world today call for a fundamental consideration of purpose—what are the prevailing influences in the world, and are they contributing to humanity's thriving in the present and far into the future? As conceived by Klein and Thompson (2025), abundance as a philosophical frame provides the opportunity to activate a different perspective, one that could include conflicting points of view, but also lead to the resolution of real problems that impact everyone's quality of life. The ability to shift to abundance relies on protecting and strengthening institutions that contribute to shared welfare or creating them where they presently do not exist. Healthy institutions are central to rebalancing a politics of power and authority with the greater affirmation made possible through reliance on values that lift up shared benefit, often reflected in grass‐roots movements at the community level.

### 5.3 Momentum

Of course, those who have culled through research and practice, drafted and redrafted, and advocated the use of the *General Principles for Leadership Programs* (ILA 2021) want them to be used. There has been selective uptake of these ideas, but much more will be required in order for progress and momentum to be possible. The prospect of ILA member communities adopting the *General Principles*, applying them in practice, and incorporating them into ongoing considerations would do three things. First, it would engage a broader cross‐section of ILA members in using and refining the *General Principles*. Second, it would provide a purpose that could relate across groups and would enhance the interdisciplinarity and cross‐cultural strengths of ILA's membership. Third, the *General Principles for Leadership Programs* (ILA 2021) could be a conduit for pursuing shared work with other organizations and institutions that place high value on good leadership and are working to build their capacity in doing so. The momentum created by these three dynamics then contributes to a final, and perhaps most important, goal—embracing together that there is much we are learning but still need to know about leadership in a changing world. By seeking the synergies of disparate perspectives and unique talents, leadership educators would be able to test, learn, and commit to ongoing refinement of what we know for the improvement of the broader world community.

## 6 Conclusion

Insights drawn from the popular author Malcolm Gladwell, with his innovative analyses of readily available data, could potentially relate to the challenges and opportunities of the leadership industry. Citing unrecognized patterns he identified in his early work, Gladwell (2024) analyzed how epidemics can be destructive but, alternatively, how epidemic strategy could be launched for the good. Applying these concepts to leadership learning, an epidemic would involve cultivating a critical mass of advocates for change, identifying superchargers, and creating a new overstory. How would that work?

Gladwell (2024) cited the research of Rosabeth Moss Canter in relation to creating welcoming work environments, where she found that a critical mass of at least 25% representation had to be present in order for any identifiable subgroup to feel welcomed and empowered. When dominant cultural group forces saw they were losing proportion, attempts to keep others below the 25% level of significance became a source of resistance. To push forward in advocating an idea and/or gaining momentum, superspreaders become critical. In the example of the COVID‐19 pandemic, superspreaders were individuals or groups that possessed some unique attribute that resulted in wide and quick spread of the virus. A new overstory is important because, if the spread of an idea or initiative is desired, the old story that restricted adoption has to be rewritten.

Klein and Thompson (2025) offered a sobering analysis of the failures of liberalism and conservatism in politics and proposed that a new story can now be defined, largely because of the failures we witness every day. Instead of political pathology that causes us to defend anything our enemies attack, a philosophy of abundance would focus on questions that matter to everyone—“What is scarce that should be abundant? What is difficult to build that should be easy? What inventions do we need that we do not yet have?” (Klein and Thompson 2025, 216).

If those of us committed to improving leadership capacity were to adopt a positive epidemic (Gladwell 2024) strategy and commit to solving bigger questions of the human condition with an abundance perspective (Klein and Thompson 2025), a new story could unfold. The new story would be informed by the previous work of many dedicated leadership scholars and educators, and it would serve as a bridge to invite others to join in the effort.

The ILA *General Principles for Leadership Programs* (ILA 2021) can be a project where a critical mass, energized by superspreaders who agree to the need to move forward in inclusive and innovative ways, can make a real difference. With economic, social, and political change so pronounced, there is little question that there is work to be done, and that it must be done more effectively. The ILA *General Principles for Leadership Programs* (ILA 2021) and the ideas they propose provide a pathway to truly create a world where leadership capacity and human creativity are abundantly released to address global challenges.
